# Cellular Energy Crisis: Particulate Hitchhikers Damage Mitochondria

**Published:** 2004-10

**Authors:** Bob Weinhold

One of the body’s most important processes—energy production by mitochondria in the cell—can be significantly disrupted by exposure to ultrafine particulates, according to a team of researchers from the University of California, Los Angeles, and the University of Southern California **[*EHP* 112:1347–1358]**. Furthermore, the researchers say, the primary culprits are substances that are attached to particles. These findings provide the first insights into the specific mechanism by which ultrafine particles, increasingly recognized as environmental villains, damage mitochondria, says principal investigator Andre Nel.

The researchers conducted a series of experiments that evaluated the effects on mouse liver mitochondria of either diesel exhaust particles (DEPs), ambient ultrafine particles collected in the Los Angeles area, or engineered nanoparticles with no attached chemicals. Using the DEPs and ultrafines collected in Los Angeles, the team isolated organic “hitchhiker” substances such as polycyclic aromatic hydrocarbons (PAHs) and quinones that had attached to the particle cores. These chemicals were tested for a variety of effects on cells and mitochondria.

Among the adverse effects observed were mitochondrial structural decomposition, mitochondrial swelling due to increased membrane porosity and rupturing, increased production of free radicals, and induction of cellular death. Although some of these effects were dependent on the presence of calcium, others were caused by direct damage to the mitochondrial membrane.

The mitochondrial effects varied with the specific hitchhiker substances tested. For instance, polar fractions high in quinones were much more potent in inducing cell death, whereas aromatic compounds high in PAHs had a more moderate effect, and aliphatic compounds had no apparent effect.

Even within a class of compounds, not all substances proved to be equally destructive. For instance, among quinones, phenanthraquinone and 1,2-naphthoquinone caused mitochondrial swelling, while anthraquinone did not. This difference may depend on the ability of particular quinones to participate in reactions that generate reactive oxygen species (ROSs), unstable compounds that can quickly react with and damage other substances.

The team speculates on the biological mechanism behind the observed effects, laying the groundwork for future research. In the case of quinones, they suggest that the substances may redirect electron transfers in the inner mitochondrial membrane to molecular oxygen, thereby generating ROSs that can damage the mitochondria as well as exert proinflammatory effects. These effects could be important in the exacerbation of asthma.

Regardless of the specific mechanism, the consistent culprits in damaging mitochondria were the organic substances attached to particle cores. In contrast, engineered polystyrene nanoparticles with no organics attached had no apparent effects, leading the team to speculate that the small size of engineered nanoparticles may not be solely responsible for inducing mitochondrial and cellular damage.

This is of considerable interest to the burgeoning field of nanotechnology, where there is concern that nanoparticles may be toxic based on small size alone. The researchers acknowledge that smaller particles tend to penetrate better than larger particles and possibly are more bioavailable, due to their higher surface-to-volume ratio. These characteristics may allow organic chemicals that are attached to particle surfaces to better penetrate tissues than if they are not carried along by tiny transporters.**–Bob Weinhold**

## Figures and Tables

**Figure:**
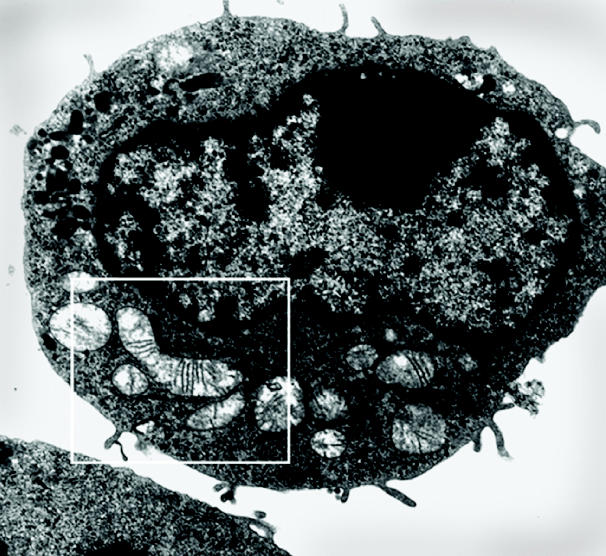
**Energy drains.** New research conducted on mouse liver cells shows that toxicants that “hitchhike” on particulates severely interfere with the ability of mitochondria (framed, above) to produce energy.
**Source:** Li N, Sioutas C, Cho A, Schmitz D, Misra C, Sempf J, et al. Ultrafine particulate pollutants induce oxidative stress and mitochondrial damage. Environ Health Perspect 111:455–460 (2003).

